# Functional Characteristics of a Highly Specific Integrase Encoded by an LTR-Retrotransposon

**DOI:** 10.1371/journal.pone.0003185

**Published:** 2008-09-11

**Authors:** Babacar Faye, Frederick Arnaud, Eric Peyretaillade, Emilie Brasset, Bernard Dastugue, Chantal Vaury

**Affiliations:** UMR/CNRS 6247, Clermont Université, INSERM, U931, Faculté de Médecine, Clermont-Ferrand, France; Ecole Normale Supérieure de Lyon, France

## Abstract

**Background:**

The retroviral Integrase protein catalyzes the insertion of linear viral DNA into host cell DNA. Although different retroviruses have been shown to target distinctive chromosomal regions, few of them display a site-specific integration. ZAM, a retroelement from *Drosophila melanogaster* very similar in structure and replication cycle to mammalian retroviruses is highly site-specific. Indeed, ZAM copies target the genomic 5′-CGCGCg-3′ consensus-sequences. To enlighten the determinants of this high integration specificity, we investigated the functional properties of its integrase protein denoted ZAM-IN.

**Principal Findings:**

Here we show that ZAM-IN displays the property to nick DNA molecules *in vitro*. This endonuclease activity targets specific sequences that are present in a 388 bp fragment taken from the *white* locus and known to be a genomic ZAM integration site *in vivo*. Furthermore, ZAM-IN displays the unusual property to directly bind specific genomic DNA sequences. Two specific and independent sites are recognized within the 388 bp fragment of the *white* locus: the CGCGCg sequence and a closely apposed site different in sequence.

**Conclusion:**

This study strongly argues that the intrinsic properties of ZAM-IN, ie its binding properties and its endonuclease activity, play an important part in ZAM integration specificity. Its ability to select two binding sites and to nick the DNA molecule reminds the strategy used by some site-specific recombination enzymes and forms the basis for site-specific integration strategies potentially useful in a broad range of genetic engineering applications.

## Introduction

Integration of the retroviral DNA genome into host-cell DNA is an essential step in the retrovirus replication cycle, permitting viral genomes to become permanently fixed as proviruses into the DNA of the host and to use host transcriptional machinery for the production of viral RNA [1]. This integration is performed by an enzyme called integrase encoded by the retrovirus. Although their mechanism of action is not yet clearly elucidated, retroviral integrases have been shown to carry out all the steps known to be required for processing and joining of the viral DNA [2]. Hotspots of integration exist and these preferences appear to be specific to the individual viruses [3]. Several studies indicate that the intrinsic properties of integrases participate in this selection. For instance, *in vitro* experiments show that integrases from different retroviruses each display a distinct and unique choice of integration sites when given an identical target DNA [4], [5]. Further experiments also indicate that local DNA sequence can influence the choice of the target site [6]. Indeed, some insertions have been associated with palindromic consensus centred on the virus-specific duplicated target site sequence, or as intrinsically bent DNA [7]. By analysing a number of sequences from HIV-1, avian sarcoma-leukosis virus (ASLV) and Murine Leukaemia Virus (MLV) into human cellular DNA, a symmetrical base preference surrounding HIV-1 and ASLV integration sites has been found [8]. Weak palindromic consensus sequences have also been reported to be a common feature at the integration target sites of many retroviruses [9]. Therefore, local DNA structure can affect insertion specificity but several studies also revealed that the chromatin structure imposed by nucleosomes or by other proteins can influence the efficiency of insertion into a particular target. Some of these proteins can be involved in chromatin structure [10]–[12], in transcription activity of nearby genes [13] or be cellular targeting proteins [4], [5]. Several cellular DNA binding proteins have been described that bind integration complexes and/or facilitate integration, including BAF, HMGa1, Ku, and LEDGF [4], [14]. Overall, despite some preferences, a high DNA sequence specificity for retroviral integration has never been described so far.

LTR-retrotransposons replication cycle is very similar to the retroviruses one. They encode *gag*, *pol* and a subclass of them have an additional *env* gene. Like retroviruses, *pol* encodes protease, reverse-transcriptase, and integrase proteins essential for retrotransposition. Various degrees of bias for the integration target sites *in vivo* have been described for these elements. The yeast *Saccharomyces cerevisiae* contains several well-studied retrotransposons –Ty1, Ty3 and Ty5- that display interesting patterns of target site selection [15], [16]. For instance, Ty1 targets the upstream sequences of transfer RNA (tRNA) or other PolIII transcribed genes [17]. Ty3 copies are also found in these regions but at a more precise location, 1–4 bp from the transcription start site [18]. This targeting is achieved by the interaction of Ty3 preintegration complex (PIC) with the PolIII transcription factor TFIIIB/TFIIIC [19]. Instead, Ty5 integrase interacts with the transcription silencing protein Sir4p and specifically targets transcriptionally ‘silent’ regions of the yeast genome, such as telomeres or the silent mating loci HM [20]–[22]. Overall, data from retroviruses and LTR-retrotransposons demonstrate a combined involvement of the Integrase, the DNA sequence and cellular host proteins to direct integration at the desired genomic DNA sites.

ZAM is an LTR-retrotransposon of 8,435-bp present within the genome of Drosophila melanogaster [23]. On the basis of sequence, structural, and functional similarities, ZAM displays a striking resemblance to vertebrate retroviruses [24]. Its three open reading frames gag, pol, and env are surrounded by two long terminal repeats or LTRs. The ZAM *pol* gene is subdivided into three regions, which encode typical retrovirus-like enzymes: protease, reverse transcriptase-RnaseH, and integrase (IN) [25], [26]. The latter displays all the characteristics of canonical retroviral IN [27]. In a previous paper, we reported that ZAM is highly sequence specific in its integration, much higher than any other retrovirus described so far. By exhaustive analyses of ZAM insertions, we have shown that the target sequence chosen by nearly every ZAM element is CGCGCg (lowercase “g” indicates a 50% occurrence of that base) [25]. However, the mechanism of this integration process and the reason of its specificity had not been elucidated.

In this paper, we investigated the functional properties of ZAM integrase in order to understand the determinant of this specificity. We investigated its endonuclease property and show that ZAM integrase cleaves specifically a genomic site known to be a target of ZAM integration *in vivo*. Our results further indicate that ZAM-IN recognizes and binds two distinct DNA sites, the CGCGCg sequence corresponding to the ZAM integration site, and a second site located in the vicinity. Our data strongly argue that ZAM-IN is the main actor in the site specificity of ZAM integration.

## Results

### The ZAM integrase displays an endonuclease activity on specific DNA fragments

Two reactions catalyzed by the integrases encoded by mammalian retroviruses have been well described: 1) the removal of two bases from the 3′ end of each viral DNA strand, and 2) the covalent attachment of leaving recessed 3′ hydroxyl groups at the viral DNA termini to protruding 5′ phosphoryl ends of host cell DNA (for review [2], [28]). Moreover, the ability of retroviral integrases to recognize, cleave and drive retroviral integration into specific DNA targets has not yet been reported although some preferences for certain genomic sites might be explained by intrinsic properties of the integrases.

**Figure 1:**
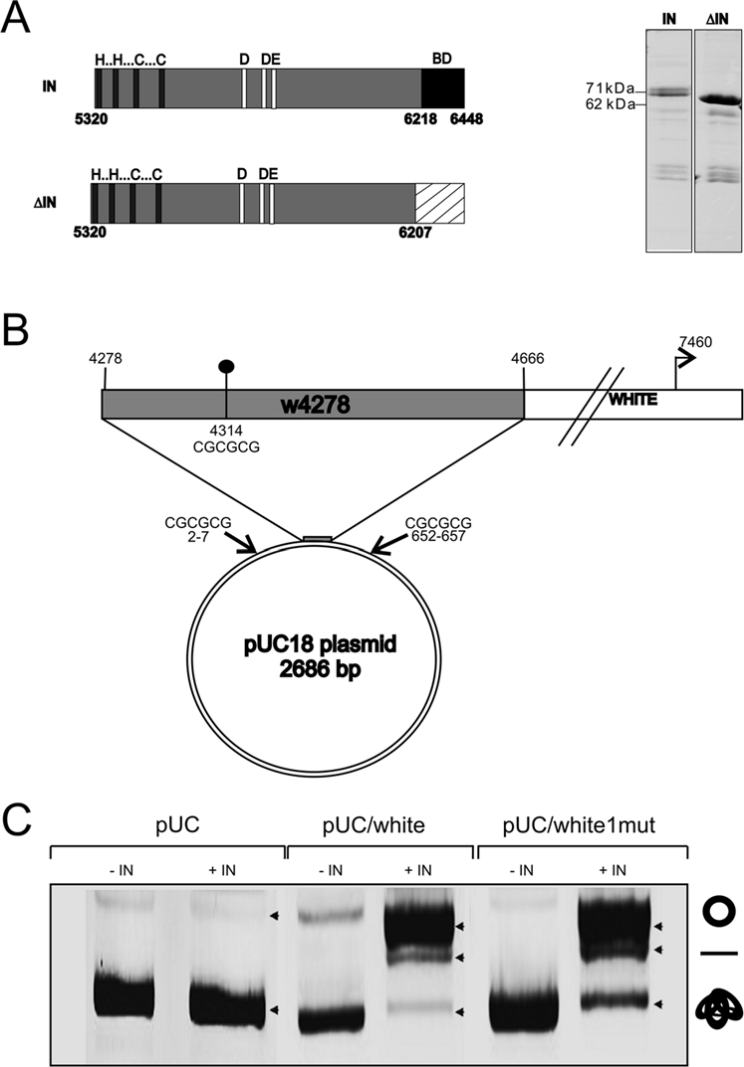
The endonuclease activity of ZAM integrase correlates with the presence of a 388 bp fragment from the *white* locus. A) Schematic representation of the ZAM integrase “IN” and a carboxy-terminal deleted integrase “ΔIN” used in the *in vitro* DNA binding assay. The three main domains: the zing finger “HHCC”, the catalytic domain “DDE” and a predictive DNA binding domain “BD” are represented. Nucleotide numbers according to ZAM sequence are indicated below. The hatched box indicated the region deleted to generate the ΔIN protein. The full length and the truncated integrases were expressed in bacteria as GST fusion proteins and fixed on agarose beads. IN and ΔIN purified proteins were analysed on SDS-PAGE electrophoresis followed by coomassie staining (right panel). The molecular masses of proteins are indicated in kilodalton. B) Circular representation of the 2686 bp pUC18 plasmid. Palindromic sequences CGCGCG present in pUC18 are indicated. The Drosophila genomic locus known to be the target of ZAM integration and located 3 kb upstream of the *white* gene is presented above. The *white* fragment (from positions 4278 to 4666 according to the drosophila sequence) was cloned in the pUC18 plasmid and is represented by the grey box. The black dot at position 4314 indicates the CGCGCG integration site of ZAM. C) *In vitro* endonuclease activity of ZAM integrase: pUC, pUC/white and pUC/white1mut plasmids were incubated without (−IN) or with (+IN) purified ZAM-IN. Positions of the supercoiled, nicked (circle) and linear (bar) DNAs are indicated.

Since ZAM copies are found integrated in a very specific consensus sequence CGCGCg, we investigated if ZAM-IN properties could explain such a targeting of the host DNA. Thus, ZAM-IN was expressed in bacteria as a GST-fusion protein and fixed on glutathione (GSH)-agarose beads (Fig. 1A, “IN”). Then we examined whether ZAM-IN intrinsic properties display a specific endonuclease activity. To this end, its endonuclease activity was assayed by measuring the ability of the purified ZAM integrase to convert supercoiled plasmids into circular and linear molecules. Experiments were conducted with two types of plasmids. One corresponds to the pUC18 cloning vector containing no insert. This plasmid displays two distinct CGCGCG sites present at nucleotide positions 2–7 and 652–657 (Fig. 1B). The second plasmid corresponds to the pUC18 vector containing a 388 bp genomic fragment taken from the upstream region of the *white* gene. This genomic fragment called w4278 from the genomic position of its 5′ end, comprises a unique consensus sequence CGCGCG (position 4314) previously described as a target for ZAM insertions [25] (Fig. 1B). Both plasmids were called pUC and pUC/white respectively. When the endonuclease activity of ZAM-IN was assayed on the pUC plasmid (see Materials and Methods), a heavy band corresponding to supercoiled molecules, and a very faint band corresponding to open circle molecules were observed for both treated and non-treated plasmids (Fig. 1C). The open circle molecules observed in ZAM-IN treated and non treated samples indicate that this population of circularized molecules resulted from DNA nicks which randomly occurred probably during DNA extraction. These results indicated that ZAM integrase is unable to cleave the pUC18 vector sequence despite the presence of two CGCGCG sites.

By contrast, an increase of open circles is clearly observed on the gel when pUC/white is incubated with ZAM-IN (Fig. 1C). This increase is easily registered between treated and untreated samples although circularized pUC/white molecules are initially present in the pUC/white DNA sample before the ZAM-IN treatment (see line 3, Fig. 1C). Thus, a nicking property of the integrase protein is registered when the *white* fragment is added to the pUC vector. In this set of assays, an increase in linear molecules that likely derive from double strand breaks generated by the ZAM-IN is also observed. However, it must be noticed that the amount of linear molecules in this experiment was higher than generally observed in other similar experiments.

Since in the same experimental conditions the pUC/white is cleaved unlike the pUC vector, it is very unlikely that the nicking property results from the activity of the purified ZAM-IN and not from the activity of a bacterial enzyme which would have been co-purified with ZAM-IN.

Overall, these results bring evidence that ZAM-IN does not nick any DNA fragment but selects and cleaves only some of them. The 388 bp *white* fragment added to the pUC plasmid carries all the signals required to drive this specific recognition ending by cleavage. Importantly, even if the CGCGCG sequence is the target site for ZAM integration, its presence is not sufficient for cleavage. This is clearly demonstrated by the fact that the pUC plasmid is not cleaved despite the presence of two CGCGCG sites. Furthermore, in an additional series of assays presented Figure 1C, we found that ZAM-IN retains the ability to cleave a plasmid named pUC/white1mut in which the CGCGCG sequence of the *white* fragment was disrupted by mutagenesis and replaced by AGAGCG. Therefore, the signal required for the endonuclease activity of ZAM-IN is not the sole CGCGCG site of the *white* fragment identified as the ZAM integration site.

### ZAM integrase binds two genomic sites within the target locus

Since an unidentified signal might exist in the 388 bp *white* fragment for the integrase to cleave the DNA, we hypothesized that some specific binding sites for ZAM-IN might be such signals.

**Figure 2:**
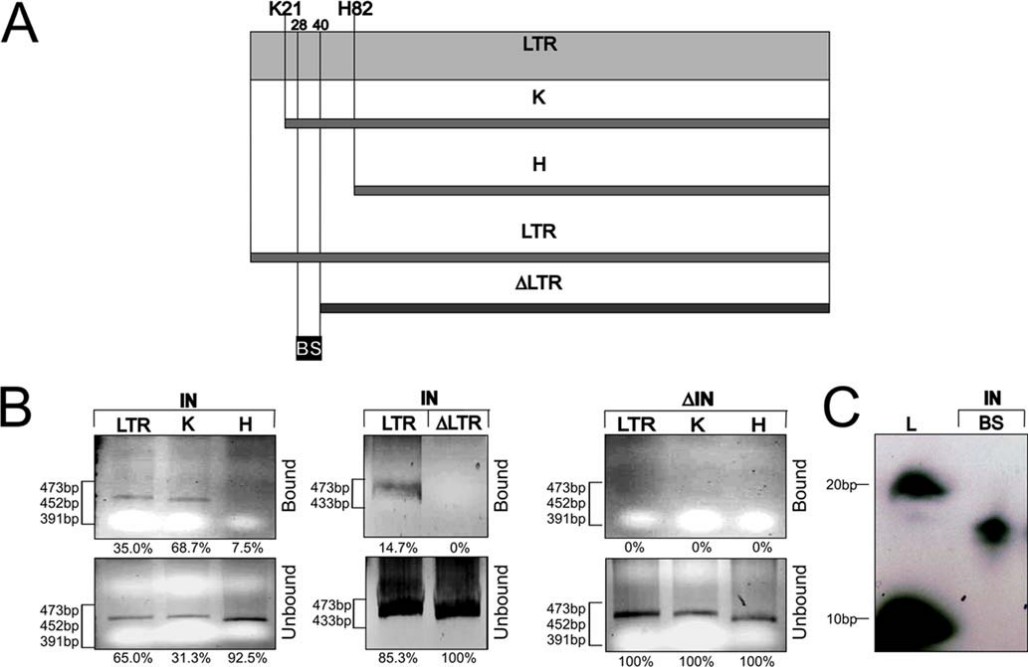
LTR binding property of ZAM integrase. A) ZAM LTR fragments. The grey box represents the full length LTR of ZAM and the solid bars represent the KpnI “K” and HindIII “H” restriction sites at the position 21 and 82, respectively. A full length LTR and truncated PCR product deleted of the first 40 bp of ZAM LTR called “ΔLTR” are presented below. A double stranded oligonucleotide spanning from position 28 to 40 and called “BS” was also used in these experiments. B) *In vitro* DNA binding assays with ZAM-IN on the LTR fragments. Left panel: The full length “LTR” and the two truncated LTR fragments digested by KpnI “K” or HindIII “H” were tested as indicated above each lane. Middle panel: the full length LTR and a truncated PCR product “ΔLTR” were used in these assays. Right panel: *In vitro* binding assays with ΔIN on ZAM LTR fragments: the full length “LTR” and the two truncated LTR fragments digested by KpnI “K” or HindIII “H” were tested as indicated above each lane. C) *In vitro* DNA binding assays performed with a double stranded oligonucleotide from base 28 to 40 according to ZAM sequence. The 13 bp fragment is retained by ZAM-IN. DNA fragments sizes are indicated as “L”. DNA fragments sizes are indicated for each panel. In B and C, bound and unbound fractions are presented in upper and lower panels respectively. The percentage of bound and unbound fractions is presented below each panel in B. In C, 100% of the 274 bp fragment was recovered in the unbound fraction whereas 100% of the 66 and 48 bp fragments were recovered in the bound fraction.

It is well demonstrated that retroviral Integrases have the property to bind each extremity of the viral DNA within their LTR sequence [29], [30]. Thus, in a first series of experiment we verified that ZAM-IN has also the capacity to bind the ZAM LTR. *In vitro* DNA binding assays were performed on the full length 5′LTR of ZAM and on two shortened LTR fragments. The GST-fusion protein of ZAM-IN fixed on beads and depicted figure 1A was used in these experiments. The LTR fragment denoted “K” corresponds to the ZAM LTR digested by KpnI to delete 21 bp from its 5′ end. The second denoted “H” corresponds to the LTR digested by HindIII to delete 82 bp from its 5′ end (Fig. 2A). As shown figure 2B, ZAM-IN is able to bind the full length LTR (upper left panel, first lane) as well as the K fragment. However, the H fragment deleted for 82 bp of ZAM 5′ end is no more retained by ZAM-IN.

To confirm these results and better localize the region recognized by the integrase, two PCR fragments corresponding to the full length or a 40 bp deleted LTR called ΔLTR were amplified and used in the same *in vitro* DNA binding assays (Fig. 2A). The results indicated that ZAM-IN is unable to retain the ΔLTR (Fig. 2B, middle panel). ZAM-IN contains three domains: a zinc finger amino-terminal motif (HHCC), a core or catalytic domain characterized by the DD35E motif, and a carboxy-terminal part of the protein which displays a high basicity similar to the DNA binding domain of retroviral integrases [25]. In order to test whether this basic domain at the C terminal end of ZAM integrase is important for its LTR DNA binding activity, this integrase deleted for its last 80 (ΔIN) was produced as a GST fusion protein and fixed on agarose beads (Fig. 1A, “ΔIN”). Then, similar *in vitro* DNA binding assays were performed with this deleted integrase ΔIN. As shown figure 2B (right panel), the ΔIN protein does not bind any of the LTR, K or H fragments of ZAM (upper panel). This result indicates that the C-terminal part of ZAM-IN is required for its binding property on ZAM LTR. It must be noticed that this experiment also confirms that the binding properties observed in the first set of experiments are indeed due to the integrase “IN” expressed *in vitro* and not to any non-specific binding. To go further, a double strand oligonucleotide labelled with [γ-^32^P]ATP and spanning nucleotide positions 28 to 40 of the 5′LTR was assayed. As shown figure 2C, this oligonucleotide called BS is retained by the Integrase. Altogether, these results indicate that ZAM-IN is able to bind the LTR of ZAM in a 13 bp site located between nucleotides 28 and 40.

**Figure 3:**
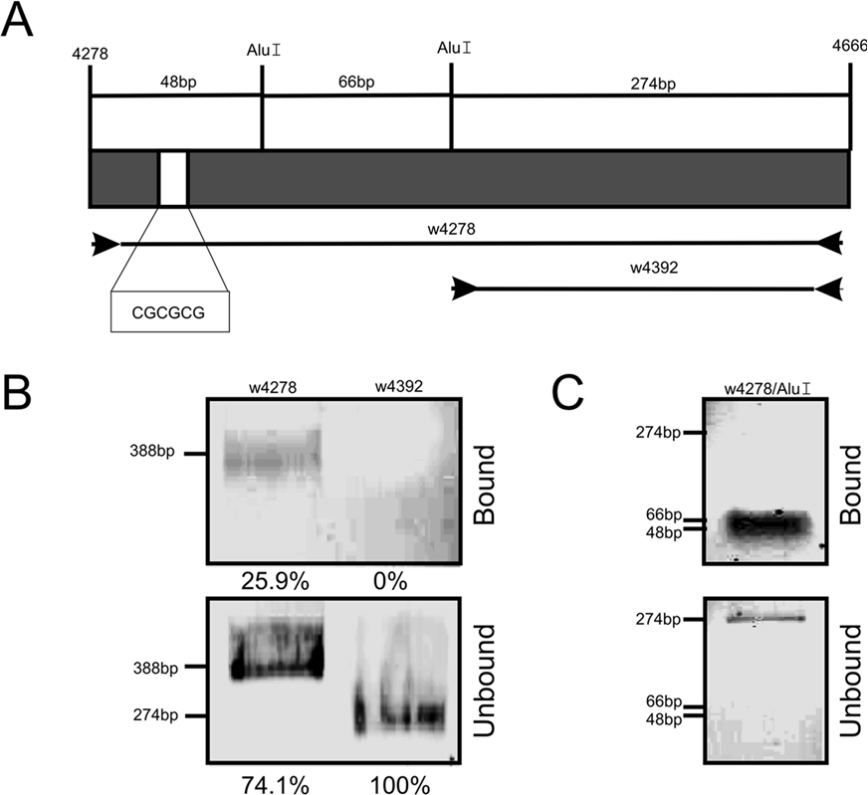
ZAM Integrase interacts with specific genomic DNA sequences. A) Diagram of the *white* DNA fragment from nucleotide positions 4278 to 4666. Two PCRs products used in this experiment and called “w4278” and “w4392” are represented underneath. The two AluI restriction sites and the resulting DNA fragment sizes are presented above. The palindromic cleavage site CGCGCG is indicated by a white box. B) *In vitro* binding assays with ZAM-IN protein performed on the *white* PCR products “w4278” and “w4392”. C) Assays performed with the *white* PCR product w4278 digested by AluI. The percentage of bound (upper panels) and unbound (lower panels) fractions is presented below each panel. DNA fragments sizes are indicated for each panel.

In a second set of experiments, we addressed whether specific binding sites for ZAM-IN exist in the 388 bp *white* fragment which could help to the target site recognition. Through *in vitro* binding assays, we searched for ZAM-IN binding sites along the 388 bp DNA fragment of *white* (Fig. 3A).

The GST-IN fusion proteins bound on beads were incubated with a PCR-amplified w4278 fragment (Fig. 1B and 3A). As shown figure 3B (lane w4278), the w4278 fragment is retained by the ZAM-IN (upper panel). This result indicates that ZAM-IN directly binds at least one DNA sequence within the 388 bp of the *white* locus.

Then, we tested whether this binding might occur indifferently along the whole length of w4278 or whether some specific binding sites could be identified. A PCR product called w4392 corresponding to a 274 bp fragment spanning from nucleotide positions 4392 to 4666 was used for further *in vitro* binding assays (Fig. 3A). As shown in figure 3B (lane w4392), this fragment is not retained by the integrase (upper panel) while it is recovered in the supernatant (lower panel). Thus, ZAM-IN is unable to bind the *white* sequence between nucleotides 4392 and 4666.

To further analyze the DNA fragment comprised between nucleotides 4278 and 4392, the PCR-amplified fragment w4278 was digested by the AluI restriction enzyme to generate three DNA fragments of 48, 66 and 274 bp long (Fig. 3A), and *in vitro* binding assays were performed. As illustrated figure 3C, the 48 bp and 66 bp fragments are retained by ZAM Integrase (upper panel). By contrast and as expected, the 274 bp fragment is not recognized by ZAM-IN and is only recovered in the supernatant (Fig. 3C, lower panel). These results indicate that ZAM-IN does not bind DNA in a random manner but has the property to recognize specific sequences. Such binding sites are located within the 48 bp and 66 bp fragments of the *white* fragment analyzed.

**Figure 4:**
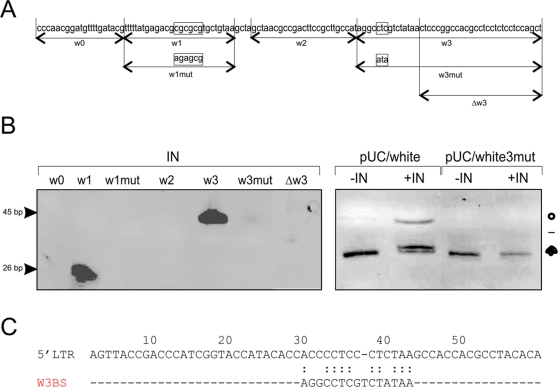
ZAM integrase binds two specific genomic DNA sites. A) Sequence of the Drosophila *white* locus from base 4278 to 4326. The oligonucleotides w0, w1, w1mut, w2, w3, Δw3 and w3mut used in the experiments are represented under the sequence. The integration site CGCGCG, the sequence of the mutated integration site of the w1mut oligonucleotide and the nucleotides mutated to generate the w3mut oligonucleotide are indicated by boxes. B) Left panel: *In vitro* binding assays were performed with ZAM integrase “IN” and the double stranded oligonucleotides w0, w1, w1mut, w2, w3, Δw3 and w3mut. w1 and w3 are the only two oligonucleotides retained by ZAM integrase. Right panel: *In vitro* endonuclease activity of ZAM integrase: pUC/white and pUC/white3mut plasmids were incubated without (−IN) or with (+IN) purified ZAM-IN. Positions of the supercoiled, nicked (circle) and linear (bar) DNAs are indicated. C) Alignment of a conserved motif detected in the ZAM LTR and w3. The first 60 nucleotides of the LTR sequence are presented as the upper sequence. The binding site of w3 is presented below.

To go further in their identification, we performed a new set of *in vitro* binding assays using four oligonucleotides encompassing the full length 48 and 66 bp fragments retained by the Integrase. Their respective positions and sequences are presented figure 4A. These oligonucleotides, called w0, w1, w2 and w3, are 21, 26, 26 and 45 nts long respectively (Fig. 4A). They were firstly annealed to complementary oligonucleotides to form double-stranded DNA molecules (see material and methods) and then used for the DNA binding assays. As illustrated figure 4B, the double-stranded oligonucleotides w1 and w3 are retained by ZAM Integrase whereas w0 and w2 are not.

Since ZAM insertion site CGCGCg is present within w1, this sequence was likely a binding site of ZAM-IN. To test this possibility, we used an oligonucleotide called w1mut in which the CGCGCG sequence was replaced by an AGAGCG site (Fig. 4A). As shown figure 4B, ZAM integrase is then unable to bind the w1mut oligonucleotide.

w3 does not contain any CGCGCG site. Thus, to identify the binding site of ZAM-IN within the w3 fragment, we tested its ability to bind diverse deleted w3 oligonucleotides. We found that when w3 is deleted for 15 bases from its 5′ end (Δw3), ZAM integrase is not able to bind the remaining sequence (Fig. 4B). Thus a second binding site of ZAM-IN is located within the first 15 bp of w3, a region in which little to no sequence similarity with the target site of integration can be detected. When analyzing the sequence of this 15 bp fragment, we detected a palindromic sequence: AGGCCT. Since the target site CGCGCG is also a palindrome, we hypothetized that some specific DNA structures such as hairpin might be recognized by ZAM-IN. A mutated oligonucleotide w3mut in which the palindromic sequence was disrupted was then tested in the same set of *in vitro* binding assays. As shown in Fig. 4B, ZAM-IN is then unable to bind w3mut. We then performed a new series of experiments similar to experiments presented Fig. 1C and assayed the endonuclease activity of ZAM-IN on a pUC/white3mut plasmid in which the 388 bp fragment of *white* displays a mutation affecting the second binding site AGGC**CTC**GTCTATAA converted to AGGC**ATA**GTCTATAA. We found that whereas ZAM-IN is able to convert supercoiled pUC/white molecules to open circles, it is unable to cleave the supercoiled molecules of the pUC/white3mut plasmid. Open circles molecules were not detected after the Integrase treatment (Fig. 4B, right panel). This result contrasts with what has been observed when the CGCGCG motif of the *white* fragment is mutated (Fig. 1C, lane pUC/white1mut). Indeed, ZAM-IN retains the ability to cleave a plasmid in which the *white* integration site is mutated whereas this ability is lost when the second binding site is destroyed.

Overall, the above experiments show that the *white* fragment necessary for the endonuclease activity of ZAM-IN displays two distinct binding sites for ZAM-IN: one of them is the integration site itself CGCGCg and the second displays a different sequence located 40 to 56 bp apart.

## Discussion

The retrotransposon ZAM displays an extreme bias in target site selection. Indeed, it integrates in a consensus sequence CGCGCg. On the basis of sequence similarity and gene organization ZAM is a member of a group of retrotransposons that bears a striking resemblance to the vertebrate retroviruses. Its enzymes involved in reverse transcription and integration are similar to retroviruses [25], [26]. Direct binding of retroviral Integrases on their LTR has been well demonstrated and we also showed that ZAM integrase binds its own LTR. However, the specific binding of retroviral IN on the DNA target sites had not been reported yet. So far, only models, in which tethering of integration machinery to host DNA via protein-protein interaction were proposed to be important for integration site selection [31]–[33], and indeed, mechanisms based on tethering strongly explain targeting of some Integrases [8], [20]. Moreover, a clear consensus motif has never been determined despite studies that highlight the influence of the primary DNA sequence in the choice of retroviral integration [7]. Our results indicate that ZAM-IN clearly binds the host DNA, and suggest that the target sites for ZAM integrations are selected through direct interaction between the target DNA and the Integrase. One binding site corresponds to the consensus CGCGCg identified as the integration site, and a second binding site with a different sequence is located in close proximity. Although the DNA characteristics of this second binding site remain to be identified, our data clearly demonstrate that if absent, ZAM-IN is unable to cleave the CGCGCg consensus site. When comparison between these two binding sites identified in the *white* fragment and the LTR fragment bound by ZAM-IN have been made, some homology was clearly detected between the second binding site of w3 and a motif located between nucleotides 30 and 45 of the LTR (Fig. 4C). Interestingly, the mutation converting the CTC triplet to ATA in w3mut and abrogating ZAM-IN binding is encompassed within this homologous site (see Fig. 4A and C). Thus, although the CGCGCg integration site is a palindromic sequence, we believe that selection of the second site might not necessarily implicate the presence of a palindrome but rather implicate constraints brought by the DNA structure or conformation. A genomic DNA organisation like a strong bending DNA structure could allow ZAM-IN to bind the site.

Among retrotransposons, the non-LTR element called R2 encodes a single protein with reverse transcriptase and endonuclease activities. R2 elements specifically insert into 28S rRNA genes of many animal groups. Christensen *et al.* (2005) have shown that the complete mechanism of integration involves two R2 protein subunits [34]. The first subunit binds upstream of the cleavage site and is responsible for the initial cleavage and reverse transcription step, while the second subunit binds downstream and is responsible for second-strand cleavage. Such properties are also observed for some restriction endonucleases like FokI, MboII or MlyI which bind a specific target sequence and cleave at a conserved distance from this binding site [35]. According to our results, this strategy is thus likely to be the one used by ZAM.

ZAM is generally present at a very low copy number in the lines of Drosophila melanogaster so that its mutagenic impact is low. However, we identified a line in which its transposition frequency suddenly increased and is correlated with a high copy number of ZAM. These insertions were found dispersed on the chromosomal arms [36], [37]. In this context, disruption of required cellular genes by these insertions could have meant suicide for both ZAM and its host. Nevertheless, the recent 20 to 30 copies of ZAM in this line have very little effect on the general biology of the host, and no clear sterility or decrease in the life cycle of the line could be detected. The characteristics of ZAM-IN reported here cannot alone explain the selection of target sites that do not compromise the health of its host. This observation suggests that *in vivo*, host factors might also contribute to the targeting of ZAM Integrase to safety regions of the genome. Experiments are under investigation to identify putatively tethering of ZAM-IN to host proteins having by themselves an additional specific recognition target. Preliminary results through two hybrid experiments have indicated that ZAM integrase interacts with SNR1, a protein of the SWI/SNF chromatin remodelling complex. Might chromatin remodelling complexes participate to the targeting of ZAM copies at specific genomic site? Further analyses are necessary to better understand the influence of such host factors in the specificity of ZAM integration.

Retroviral vectors, which integrate the host chromosomes, are the most widely used method of gene transfer in mammals. However, such insertions within the genome come with a cost. Insertions near cellular proto-oncogenes leading to ectopic gene activation have been seen in two patients undergoing retrovirus-based gene therapy [38]. Understanding the molecular mechanism underlying integration site selection of elements related to retroviruses such as ZAM brings the hope that some new strategy will be found to direct integration to innocuous chromosomal sites and avoid problems generated by the little target specificity of vectors currently used.

## Materials and Methods

### GST-Integrase expression and purification

Oligonucleotides ZAM5322BamHI (gaatccgatgcaaatcacttc) and ZAM6207BamHICi (ggattcctgttaggttgtact) or ZAM6448BamHICi (ggattcctaggaggttggtgc) were used to clone at the BamHI restriction site, respectively, the full length ZAM integrase or a deleted ZAM integrase peptide called ΔIN in frame with the GST protein in the pGEX-5-X1 vector (Amersham Pharmacia Biotech). BL21 transformant colonies were inoculated in 100 ml of LB/ampicillin medium and incubated over night at 37°C. Expression of both GST-IN and GST-ΔIN fusion proteins in *Escherichia coli* BL21 was induced for 4 hrs at 30°C with 0.1 mM IPTG (isopropyl-*β*-D-thiogalactopyranoside). Pellets were resuspended in 10 ml of ice-cold solubilization buffer (50 mM Tris–HCl pH 7.4, 1 mM EDTA, 100 mM NaCl, 10% glycerol, 1% NP-40, 1 mM DTT, 1 nM PMSF, 10 µg/ml aprotinin, 2 µg/ml leupeptin, 2 µg/ml pepstatin, 0.5 mg/ml lysozyme). After sonication, supernatants were incubated for 30 min with 1 ml of 50% glutathione–agarose beads, washed three times in 1 M NaCl, three times in PBS and resuspended in 1 ml of PBS. The GST-IN and GST-ΔIN proteins fixed on agarose beads were used for *in vitro* DNA binding assays. For *in vitro* endonuclease experiments, GST-IN fusion proteins were eluted from beads by incubating for 30 min at 4°C in 10 mM glutathione/50 mM Tris–HCl, pH 9.

### Constructs and Direct mutagenesis

The *white* fragment from nucleotide 4278 to 4666 (according to the accession number: X02974) was amplified by PCR on a genomic template and cloned in pUC18 giving rise to the pUC/white plasmid. From the pUC/white plasmid, direct mutagenesis (Pfu Turbo DNA Polymerase from Stratagene) of the palindromic site from CGCGCG to AGAGCG was performed with the following sense and reverse oligonucleotides: white1mut:“tttttatgagac**aa**g**a**gcgtgctgtaacct” and white1mutCi “aaaaatactctg**tt**c**t**cacgacattgga”. The resulting plasmid was called pUC/white1mut.

### *In vitro* endonuclease reactions

*In vitro* endonuclease reactions were performed as followed: 0.5 µg of 5′LTR substrate, 10 ng of purified ZAM-IN fusion protein, and 2 µg of target DNA (pUC18, pUC/white, and pUC/white1mut) were incubated in 20 µl of 20 mM Tris (pH 8.0)-0.01% bovine serum albumin-1 mM dithiothreitol -10% dimethyl sulfoxide, 2 mM MnCl_2_ and 10 mM MgCl_2_ at 30°C for 2 hour. Analysis of DNA was performed on a 1% agarose gel stained by Ethidium Bromide.

### *In vitro* DNA binding assay

The full length 5′LTR of ZAM (473 bp long) called “LTR” and a “ΔLTR” deleted for the first 40 bp were amplified by PCR using forward primers ZAM1: (agttaccgacccatcggtacc) or ZAM40: (taagccaccacgcctacacaa), respectively, and the reverse primer ZAM473CI: (agttacctccggggagtcttg). The full length LTR product was digested with either KpnI or HindIII located at positions 21 and 82, respectively, from the 5′end of the 5′LTR sequence of ZAM. The LTR and ΔLTR PCR products as well as the KpnI and HindIII digested fragments were used for *in vitro* DNA binding experiments. Moreover, a doubled stranded oligonucleotide called “BS” from base 28 to 40 according to ZAM sequence was labelled with [γ-^32^P]ATP by a kinase reaction (Invitrogen) and used in this study. The *white* locus fragments from position 4278 to 4666 and 4392 to 4666 were amplified by PCR and called w4278 and w4392 respectively. PCR products of w4278 were also digested by the AluI restriction enzyme. These two PCR products and the AluI digested fragments were used for *in vitro* DNA binding experiments. Double stranded *white* oligonucleotides: w0 (cccaacggatgttttgatacg), w1 (tttttatgagacgcgcgcgtgctgta), w1mut (tttttatgagacaagagcgtgctgtaa), w2 (agctaacgccgacttccgcttgccat), w3 (aggcctcgtctataactcccggccacgcctcctctcctccagct), w3mut (aggcatagtctataactcccggccacgcctcctctcctccagct), Δw3 (ctcccggccacgcctcctctcctccagct) were also used in these experiments. For each reaction, DNA fragments or oligonucleotides were mixed with 20 µl of GST–IN protein fixed on glutathione–agarose beads in the binding buffer [10 mM HEPES, 50 mM KCl, 1 mM DTT, 2.5 mM MgCl_2_, 20 µg/ml poly(dI–dC), 7.5% glycerol pH 7.9] and incubated at room temperature for 3 h. Beads were pelleted and washed three times in a binding buffer containing 100 mM NaCl to remove all fragments that were not tightly bound. DNA that remained bound to the beads was extracted by phenol/chloroform, precipitated and resuspended in TE before being analyzed on a 1% agarose gel or a 15% polyacrylamide gel stained by Ethidium Bromide.
